# Complete Genome Sequence for the Shellfish Pathogen *Vibrio coralliilyticus* RE98 Isolated from a Shellfish Hatchery

**DOI:** 10.1128/genomeA.01253-14

**Published:** 2014-12-18

**Authors:** Gary P. Richards, James L. Bono, Michael A. Watson, David S. Needleman

**Affiliations:** a U.S. Department of Agriculture, Agricultural Research Service, Dover, Delaware, USA; b U.S. Department of Agriculture, Agricultural Research Service, Clay Center, Nebraska, USA; c U.S. Department of Agriculture, Agricultural Research Service, Wyndmoor, Pennsylvania, USA

## Abstract

*Vibrio coralliilyticus* is a pathogen of corals and larval shellfish. Publications on strain RE98 list it as a *Vibrio tubiashii*; however, whole genome sequencing confirms RE98 as *V. coralliilyticus* containing a total of 6,037,824 bp consisting of two chromosomes (3,420,228 and 1,917,482 bp) and two megaplasmids (380,714 and 319,400 bp).

## GENOME ANNOUNCEMENT

*Vibrio coralliilyticus* is a well-known coral pathogen responsible for coral bleaching and the associated losses to coral reefs worldwide (1, 2). It was also shown to infect a variety of shellfish larvae including Pacific oyster (*Crassostrea gigas*) larvae (3, 4). Another reported pathogen of Pacific as well as Eastern oyster (*C. virginica*) larvae is *Vibrio tubiashii*, which causes high mortalities in oyster and clam hatcheries and potentially in the wild (5–8). Some marine isolates thought to be *V. tubiashii*, like RE22 and the American Type Culture Collection strain ATCC 19105, have been identified by sequencing as *V. coralliilyticus* (9). Strain RE98, previously thought to be a *V. tubiashii* (8, 10), is particularly virulent toward larval oysters and clams and is identified in this paper by complete genome sequencing as *V. coralliilyticus*. A comparison of mortalities of Eastern and Pacific oyster larvae to RE98 indicate that this strain is most pathogenic among five strains of *V. coralliilyticus* tested (11). The misidentification of several *V. coralliilyticus* as *V. tubiashii* has complicated the discernment of the roles of these pathogens in larval shellfish mortalities and as potential etiological agents involved in coral bleaching.

The genome of *V. coralliilyticus* RE98 was sequenced using a PacBio RS II system (Pacific Biosciences, Menlo Park, CA) on single-molecule real-time (SMRT) cells using PacBio P5-C3 chemistry. Subread filtering was performed with the SMRT Analysis Software suite (12), error correction and assembly was conducted with Celera Assembler v8.1 (13), overlapping ends were trimmed using Geneious v7.1.5 (Biomatters, Auckland, New Zealand) and polished with Quiver (12). Coverage was 20×, and assemblies gave a consensus accuracy of 99.9996 to 100%. The fully assembled and closed genome contains 6,037,824 bp consisting of two chromosomes and two megaplasmids. Chromosome 1 is 3,420,228 bp, chromosome 2 is 1,917,482 bp, and the two megaplasmids (P381 and P319) are 380,714 and 319,400 bp, respectively. This is the first complete genome sequence for this species.

Genome annotation for *V. coralliilyticus* RE98 was acquired from the NCBI Prokaryotic Genome Annotation Pipeline (Bethesda, MD) and revealed 5,718 genes, 5,467 coding sequences, 98 pseudogenes, 34 rRNAs (5S, 16S, and 23S), 116 tRNAs, 3 noncoding RNA, and 11 frameshift genes. A Blast search of chromosomes 1 and 2 using the NCBI whole-genome shotgun (WGS) contig database, limited by organism (*Vibrionaceae*), showed >97% sequence similarity to other *V. coralliilyticus*.

### Nucleotide sequence accession numbers.

The complete genomic sequence of *V. coralliilyticus* RE98 (chromosomes 1 and 2 and its two megaplasmids) has been deposited in GenBank under accession no. CP009617, CP009618, CP009619, and CP009620.

## References

[1] Ben-Haim Y Thompson FL Thompson CC Cnockaert MC Hoste B Swings J Rosenberg E 2003 *Vibrio coralliilyticus* sp. nov., a temperature-dependent pathogen of the coral *Pocillopora damicornis*. Int. J. Syst. Evol. Microbiol. 53:309–315. 10.1099/ijs.0.02402-0. 12656189

[2] Ben-Haim Y Zicherman-Keren M Rosenberg E 2003 Temperature-regulated bleaching and lysis of the coral *Pocillopora damicornis* by the novel pathogen *Vibrio coralliilyticus*. Appl. Environ. Microbiol. 69:4236–4242. 10.1128/AEM.69.7.4236-4242.2003. 12839805 PMC165124

[3] Kesarcodi-Watson A Miner P Nicolas J-L Robert R 2012 Protective effect of four potential probiotic organisms against pathogen-challenge of larvae of three bivalves: Pacific oyster (*Crassostrea gigas*), flat oysters (*Ostrea edulis*) and scallop (*Pecten maximus*). Aquaculture 344–349:29–34. 10.1016/j.aquaculture.2012.02.029.

[4] Genard B Miner P Nicolas J-L Moraga D Boudry P Pernet F Tremblay R 2013 Integrative study of physiological changes associated with bacterial infection in Pacific oyster larvae. PLoS One 8:e64534. 10.1371/journal.pone.0064534. 23704993 PMC3660371

[5] Brown C 1981 A study of two shellfish-pathogenic *Vibrio* strains isolated from a Long Island hatchery during a recent outbreak of disease. J. Shellfish Res. 1:83–87.

[6] Hada HS West PA Lee JV Stemmler J Colwell RR 1984 *Vibrio tubiashii* sp. nov., a pathogen of bivalve mollusks. Int. J. Syst. Bacteriol. 34:1–4. 10.1099/00207713-34-1-1.

[7] Elston R Frelier PF Cheney D 1999 Extrapallial abscesses associated with chronic bacterial infections in the intensively cultured juvenile Pacific oyster *Crassostrea virginica*. Dis. Aquat. Org. 37:115–120. 10.3354/dao037115.

[8] Elston RA Hasegawa H Humphrey KL Polyak IK Häse CC 2008 Re-emergence of *Vibrio tubiashii* in bivalve shellfish aquaculture: severity, environmental drivers, geographic extent and management. Dis. Aquat. Org. 82:119–134. 10.3354/dao01982. 19149375

[9] Wilson B Muirhead A Bazanella M Huete-Stauffer C Vezzulli L Bourna DG 2013 An improved detection and quantification method for the coral pathogen *Vibrio coralliilyticus*. PLoS One 8:1–7. 10.1371/journal.pone.0081800. PMC3858260 24339968

[10] Hasegawa H Lind EJ Boin MA Häse CC 2008 The extracellular metalloprotease of *Vibrio tubiashii* is a major virulence factor for Pacific oyster (*Crassostrea gigas*) larvae. Appl. Environ. Microbiol. 74:4101–4110. 10.1128/AEM.00061-08. 18456850 PMC2446533

[11] Richards GP Watson MA Needleman DS Church KM Häse CC 24 October 2014 Mortalities of Eastern and Pacific oyster larvae by the pathogens *Vibrio coralliilyticus* and *Vibrio tubiashii*. Appl. Environ. Microbiol. 10.1128/AEM.02930-14. PMC4272748 25344234

[12] Chin CS Alexander DH Marks P Klammer AA Drake J Heiner C Clum A Copeland A Huddleston J Eichler EE Turner SW Korlach J 2013 Nonhybrid, finished microbial genome assemblies from long-read SMRT sequencing data. Nat. Methods 10:563–569. 10.1038/nmeth.2474. 23644548

[13] Koren S Harhay GP Smith TP Bono JL Harhay DM Mcvey SD Radune D Bergman NH Phillippy AM 2013 Reducing assembly complexity of microbial genomes with single-molecule sequencing. Genome Biol. 14:R101. 10.1186/gb-2013-14-9-r101. 24034426 PMC4053942

